# Supplementary material for: Absence of item origin bias on a Brazilian interinstitutional Progress Test examination: A pooled analysis of items approach
Source: PLoS One. 2025 Jun 9;20(6):e0325734. doi: 10.1371/journal.pone.0325734 (PMC12148128; doi:10.1371/journal.pone.0325734)
Supplement: Attachment — Submitted filename: POne_Answers.docx [file pone.0325734.s003.docx]

**RESPONSES TO THE REVIEWERS’ COMMENTS**

**REVIEWER #1:**

**Dear Author,**

**Thank you for the opportunity to review this manuscript. Below are the comments on my review:**

**1. The subject matter of the article is relevant to be published in the journal. However, it needs minor revisions.**

*We thank the reviewer for the thoughtful comments that certainly improved the	quality of our manuscript.*

**2. The title clearly reflects the subject matter of the article. However, it could mention that the study was conducted in Brazil, to better define the research.**

*We added “Brazil” in the title.*

**3. The abstract and keywords provide good information about the article. The subject matter, the objective of the research, the methodology used, the main results and conclusions are mentioned. However, the abstract fails to mention the objective of the research.**
*We agree that the aim was missing. We added it to the Abstract.*

**4. Check whether all authors cited in the article are in the manuscript's references, and vice versa.**
*Everything is ok with this regard.*

**5. The article has a few problems with clarity and coherence in its language, which need to be revised. The Introduction needs to make the objective of the research clearer. The methodology is clear and highlights the data generation locations, data collection, method used, study participants and data analysis method. However, it is necessary to present the research conclusions, which are intertwined with the Discussion section of the manuscript.**
*We checked English Language. Also, we stated the objective at the end of Introduction.	The Conclusions are presented now in a separate section.*

**6. There is articulation between the theme and the theoretical basis. There is a good dialogue with the international scientific literature that addresses this subject.**

*Thank you.*

**7. There is data analysis and coherence in the argumentation. Although there are no categories of data analysis, they are organized and analyzed, according to the method used, in dialogue with the scientific literature consulted.**

*Thank you.*

**8. The bibliography used is adequate and up-to-date in the article.**

*Ok.*

**This is an interesting study. However, I hope these comments help improve your manuscript. Congratulations on your research.**

*Thank you for appreciating our study and for taking the time to kindly review our	manuscript.*

**REVIEWER #2:**

**1.The manuscript states: "This consortium conducts biannual examinations for all students (approximately 4500) spanning the first to sixth undergraduate years." However, the analysis focuses solely on 6th-year students. If the PT is designed at the final-year level and thus cannot be extended to earlier years, the authors should explicitly justify this sampling choice.**

*Yes, your observation is absolutely correct. We have justified our choice accordingly on the Methods section.*

**2.The use of a single-test, cross-sectional dataset may fail to capture dynamic aspects of item origin bias (e.g., temporal trends or interactions with student cohorts). The authors should discuss how this limitation might affect their findings and consider incorporating longitudinal data (e.g., multiple test administrations) to verify result robustness.**

*We have discussed the limitation of using a single-test database – we now reinforced that it really may have failed to provide more robust data. We agree with the reviewer and, even though we cannot overcome this limitation in the present study, our group is adopting longitudinal efforts to verify origin bias throughout all progress test examinations. We hope that we can present the generated data on upcoming studies.*

**3.While the study controls for item origin, student performance may also be influenced by institutional factors (e.g., curriculum focus, teaching quality, or student demographics). The authors should address whether these were accounted for (e.g., via sensitivity analyses or covariates). Additionally, the superior performance of Schools B/F/G could stem from non-bias factors (e.g., selective admissions or targeted training). Exploring these possibilities would enrich the discussion.**

*We agree with the reviewer that probably there are many factors influencing the superior performance of schools B, F, and G. However, as we have stated on Ethical Considerations section, no school was disclosed in the presentation of results in accordance with the consortium’s code of conduct. It means that not only the schools’ name would be published but also that one school will not know the performance of the students from others. We are quite aware that PT allows for cross-institutional benchmarking – however, these comparisons are made only on private sessions. We will take the reviewer’s comments to study the viability of investigating how the aforementioned factors may cause differences on PT scores, on the basis of the school’s directors’ approval. And finally, we understand that controlling for other institutional factors goes beyond the scope of the present investigation.*

**4.The rationale for choosing pooled analysis over alternative methods (e.g., multilevel modeling) should be clarified. A brief explanation of its advantages (e.g., handling sparse data, aggregating item-level effects) would aid readers in evaluating the approach.**
*We added this explanation on the Discussion.*

**5.The random-effects model reports moderate-to-high heterogeneity (I²). To quantify this further, the authors should report Q-test p-values and explore potential sources. This would help assess whether heterogeneity undermines the "no bias" conclusion.**
*We agree with the comment. We present the Q-test results as Supplemental Material. The values were presented beside The I^2^ results.* However, we believe that high heterogeneity rates on I2 analyses suggest a non-uniform pattern of students’ responses across the exam items, indicating high variability between schools and supporting the absence of origin bias. In other words, a uniform pattern of correct answers would suggest a homogeneous pattern – possibly due to origin bias. We added this reasoning on the Discussion.

**6. The statement "no discernible differences in psychometric indices based on the school responsible for item creation" requires deeper contextualization. The authors should:
Contrast their null result with prior positive findings (e.g., Mujijtjens et al.), highlighting methodological or contextual differences. Discuss whether the absence of bias reflects true fairness or methodological constraints (e.g., limited power). Emphasize how their consortium’s practices (e.g., blueprint adherence, faculty training) might mitigate bias, offering actionable insights for other institutions.**

*We added explanation with regards to the psychometric indices across the school’s items. We expanded the Discussion and the Conclusion (as suggested by Reviewer #1) emphasizing the interest of our method and our practices at the consortium.*
